# Hyper-reticulated calixarene polymers: a new example of entirely synthetic nanosponge materials

**DOI:** 10.3762/bjoc.14.127

**Published:** 2018-06-20

**Authors:** Alberto Spinella, Marco Russo, Antonella Di Vincenzo, Delia Chillura Martino, Paolo Lo Meo

**Affiliations:** 1 CGA-ATeNCenter, Università degli Studi di Palermo, Via F. Marini 14, 90128 Palermo, Italy; 2 Dipartimento di Scienze e Tecnologie Biologiche, Chimiche e Farmaceutiche (STEBICEF), Università degli Studi di Palermo, V.le delle Scienze ed. 17, 90128 Palermo, Italy

**Keywords:** calixarenes, nanosponges, smart materials, solid-state NMR

## Abstract

New calixarene-based nanosponges (*Ca*NSs), i.e., hyper-reticulated polymers constituted by calixarene monomer units joined by means of bis(1,2,3-trialzolyl)alkyl linkers, were synthesized, characterized and subjected to preliminary tests to assess their supramolecular absorption abilities towards a set of suitable organic guests, selected as pollutant models. The synthesis was accomplished by means of a CuAAC reaction between a tetrakis(propargyloxy)calix[4]arene and an alkyl diazide. The formation of the polymeric network was assessed by means of FTIR and ^13^C{^1^H} CP-MAS solid-state NMR techniques, whereas morphological characterization was provided by SEM microghaphy. The materials were proved to possess pH-dependent sequestration abilities, due to the presence of the weakly basic triazole linkers. Sequestration efficiency indeed depends on the effective occurrence of both electrostatic and hydrophobic interactions between the guest and the polymer lattice. Thus, our *Ca*NS nanosponges can be considered as a new class of purely synthetic smart absorbent materials.

## Introduction

Nanosponges (NSs) [1–3] constitute an emerging area of both materials chemistry and supramolecular chemistry due to their peculiar properties, which have been object of an increasing interest during the last years. These materials are constituted by hyper-reticulated polymers obtained joining supramolecular host units, as the monomers, by means of a suitable reticulating agent. Up to date, the examples by far most studied are constituted by cyclodextrin-based nanosponges (*Cy*NSs) [4–5], which can be synthesized by reacting native β-cyclodextrin with double electrophiles such as epichlorohydrin [6], organic carbonates [7–9] or bis-isocyanates [10], in variable ratios depending on the required degree of reticulation. The process, of course, exploits the nucleophilic reactivity of the hydroxy groups of the macrocycle oligosaccharide unit. In general, *Cy*NSs possess an extensive random network of nanochannels and nanocavities. Despite their apparently low porosity and surface area (as determined by BET techniques) [11–13], the possibility to load them with a guest up to an almost exhaustive occupancy of the cyclodextrin host units [12] indicates that these materials are well permeable to solutions. The *Cy*NSs have been successfully tested and employed in several contexts, such as drug carrier systems [5,14–15], preservative agents delivery [16], pollutants sequestration [17–20] etc. Various uses of nanosponges have been recently reviewed [21–27]. The properties of *Cy*NSs, in terms of physical and mechanical features, porosity, thermal stability and adsorption or release abilities, may be modulated up to a certain extent by a sensible choice of the linker unit, depending on its length and possible functionalization. For instance, increased porosity of the *Cy*NS obtained has been recently claimed by the use of a rigid terephthalonitrile unit as the linker [13]. On the other hand, it has been recently shown that polyamine linkers give rise to pH-sensitive materials with tunable adsorption abilities [12].

In order to extend and possibly improve the supramolecular binding abilities of *Cy*NSs, mixed cyclodextrin-calixarene co-polymers nanosponges (*CyCa*NSs) were recently synthesized by exploiting a classical “click-chemistry” approach, namely the CuAAC reaction (Cu-catalyzed azide–alkyne cycloaddition [28–30]) between a heptakis(6-azido-6-deoxy)-β-cyclodextrin and a tetrakis(propargyloxy)calix[4]arene [11,31–32]. In this way, a random disposition of the co-monomer units linked by 1,2,3-triazole units is achieved. The obtained *CyCa*NSs benefit from several advantages. First, the properties of the material can be largely tuned by varying the combination ratio between the two co-monomers. In fact, full reticulation is achieved even though the co-monomers are not reacted in equivalent amounts. Moreover, in the latter case reactive azide or alkyne functional groups in excess are present throughout the polymeric structure and can be subsequently subjected to further chemical transformation, opening the way to a possible post-functionalization [31]. This, in turn, largely improves the possible tunability in the properties of the materials obtained. Finally, the triazole linker present in the structure is quite interesting per se, because it is a weak base [33], and thus it is able to provide fair pH-sensitivity even to non-modified *CyCa*NSs [31]. Moreover, it is also a structural motif present in various molecules of pharmaceutical interest [34–36]. It has been recently demonstrated that *CyCa*NS composites loaded with quercetin may show improved cytotoxic activity towards some human breast cancer cell lines [32], likely due to a synergistic action between the polyphenol nutraceutic guest molecule and triazole derivatives coming from the progressive disgregation of the co-polymer carrier.

**Scheme 1:**
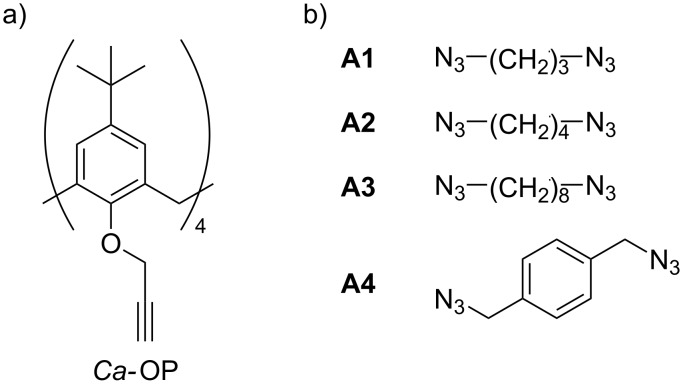
Structures of: a) calixarene *Ca*-OP; b) alkyl diazides **A1**–**A4**.

**Scheme 2:**
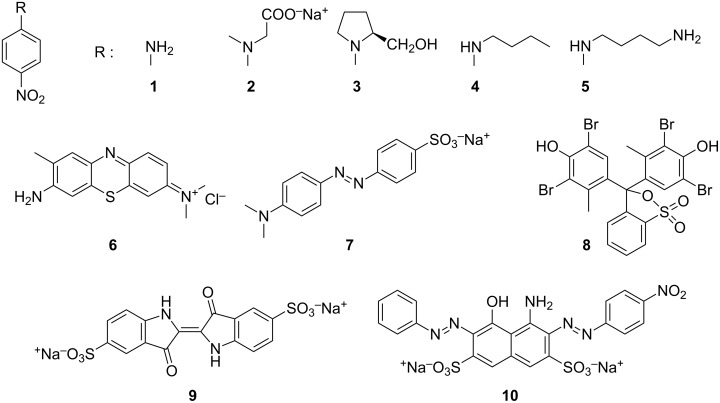
Structures of *p*-nitroaniline derivatives **1**–**5** and dyes **6**–**10**.

From the organic synthesis viewpoint, both *Cy*NSs and *CyCa*NSs are “artificial” materials, in the sense that they derive from chemical manipulation of a natural occurring starting synthon, namely the cyclodextrin unit. We reasoned that it could be very interesting to explore the possibility of obtaining an entirely synthetic NS, and testing the supramolecular binging abilities of a material exclusively constituted by calixarene units. For these purposes, in the present work we describe the synthesis, the characterization and a preliminary study on the adsorption abilities of a set of new calixarene nanosponges (*Ca*NSs), in which the host monomers are joined by means of bis(1,2,3-triazole) subunits having different features. More in detail, four different nanosponges *Ca*NS1-*Ca*NS4 were obtained by reacting the 5,11,17,23-tetra-*tert*-butyl-25,26,27,28-tetrakis(propargyloxy)calix[4]arene (*Ca*-OP, Scheme 1a) with four different alkyl diazides **A1**–**A4** (Scheme 1b), in which the two azido groups are separated by spacers having different length and rigidity. The materials obtained were characterized by means of combined spectroscopic (FTIR, solid state ^13^C{^1^H} CP-MAS NMR), calorimetric (DSC) and imaging (SEM) techniques. Then, their sequestration abilities were tested in aqueous medium at different pH values, in order to assess their possible pH-tunable properties, towards a set of structurally diverse organic guests (4-nitroaniline derivatives **1**–**5** and dyes **6**–**10**, Scheme 2) which were suitably selected as possible pollutant models.

## Results and Discussion

### Synthesis and FTIR characterization

The synthesis of materials *Ca*NS1-*Ca*NS4 was first attempted according to the procedure already optimized for the synthesis of *CyCa*NS co-polymers [11], i.e., by reacting the monomer precursor *Ca*-OP with an equivalent amount of each alkyl diazide for 18 h at 70 °C in 4.0 mL of DMSO. Unfortunately, we noticed that by applying these conditions only an intractable mixture of unidentifiable oligomers could be isolated. However, we succeeded in obtaining the desired fully reticulated polymers by reducing the amount of solvent (2.0 mL) and extending the reaction time up to 90 h. These observations can be easily explained considering that the diazides used for our purposes are much less effective reticulating agents as compared to the heptakis(6-azido-6-deoxy)-β-cyclodextrin used for the synthesis of *CyCa*NSs, simply because of the different number of azido groups present in the molecule.

**Figure 1:**
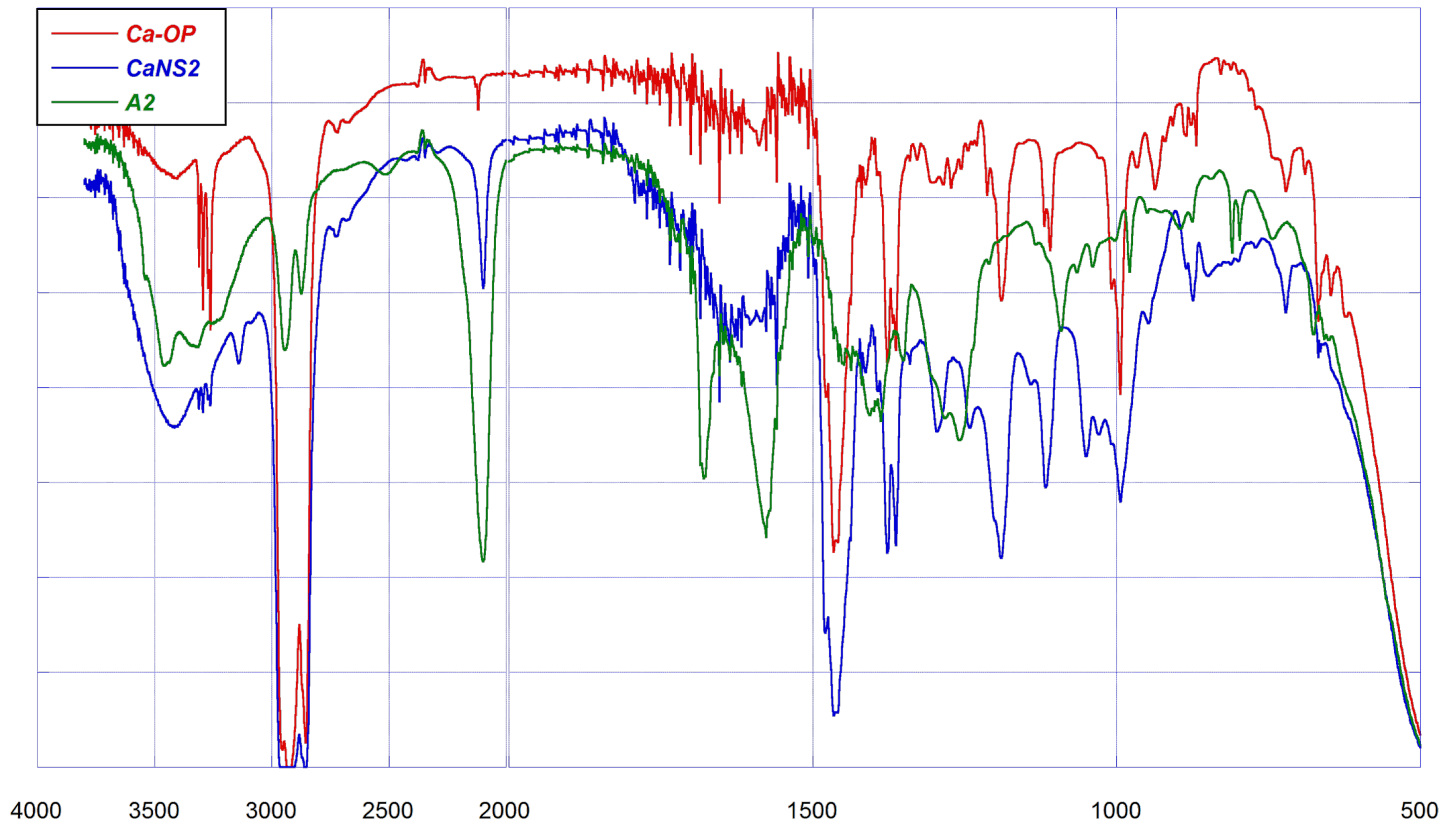
FTIR spectra of *Ca*-OP (red), A2 (green) and *CaNS2* (blue).

The actual accomplishment of the CuAAC reaction, and therefore the formation of the reticulated polymer network, was first assessed by means of FTIR spectroscopy. The FTIR spectra of the propargyloxycalixarene precursor *Ca*-OP, of the diazide **A2** and the relevant material *Ca*NS2 are shown synoptically in Figure 1 for exemplificative purposes.

The spectrum of the calixarene precursor is characterized by: i) a group of four peaks in the region 3310–3260 cm^−1^ attributable to the C_sp_–H stretching vibration; ii) a tiny signal at 2124 cm^−1^ attributable to the C≡C stretching; iii) characteristic fingerprint signals at 1478, 1363, 1191, 1109, 994, 937 and 721 cm^−1^. On the other hand, **A2** presents a strong absorption peak at ca. 2098 cm^−1^ typical of the azido group stretching, and fingerprint bands at 1406, 1387, 1351, 1258, 1092, 979, 874, 810 and 798 cm^−1^. The broad absorption bands in the region 3600–3100 cm^−1^ and at 1680 cm^−1^ are due to the unavoidable presence of water in traces. On passing to the spectrum of the product *Ca*NS2, one can first notice the presence of a new tiny band at 3140 cm^−1^, which can be attributed to a C_sp2_–H stretching. At the same time a residual presence of the C_sp_–H and -N_3_ stretching bands can be detected. These findings account for the formation of the new triazole rings upon occurrence of the CuAAC reaction. However, the presence of the residual signals relevant to the former reactive functional groups indicates that the coupling reaction does not proceed up to completion. The latter observation is perfectly consistent with what occurs in the synthesis of *CyCa*NSs, and can be easily explained considering that, due to the hyper-reticulated structure of the resulting material, a fair amount of reactive -N_3_ and -C≡CH functional groups cannot approach correctly each other to accomplish the coupling reaction. For instance, in the case of *CyCa*NSs it has been found that, even though the reactants are placed in equivalent amounts, more than 20% of functional groups do not actually react [11]. On the other hand, the actual formation of the triazole ring can be confirmed by the examination of the fingerprint region. According to literature, the presence of the heterocycle linker is accounted for by the occurrence of a tiny signal at 1242 cm^−1^ [11]; moreover, new bands are present in the spectrum of *Ca*NS2 at 1296, 1050 and 1026 cm^−1^, which cannot be attributed to either of the starting reactants. Further signals at 1480, 1364, 1190, 1117, 995 and 721 cm^–1^ are in fact signals relevant to the calixarene scaffold. As a final remark, the spectra of *Ca*NS1 and *Ca*NS3 present nearly the same spectral features, and do not need to be discussed in detail. Interestingly, in the spectrum of *Ca*NS4 the signal relevant to the residual unreacted azido groups at 2089 cm^−1^ appears relatively more intense as compared to the other materials. This can be easily justified considering that the rigid aromatic linker **A4** makes more difficult the correct approach between the reactive groups during the construction of the polymer network, as compared with the flexible linkers **A1–A3** (see below).

### Solid-state NMR characterization

**Figure 2:**
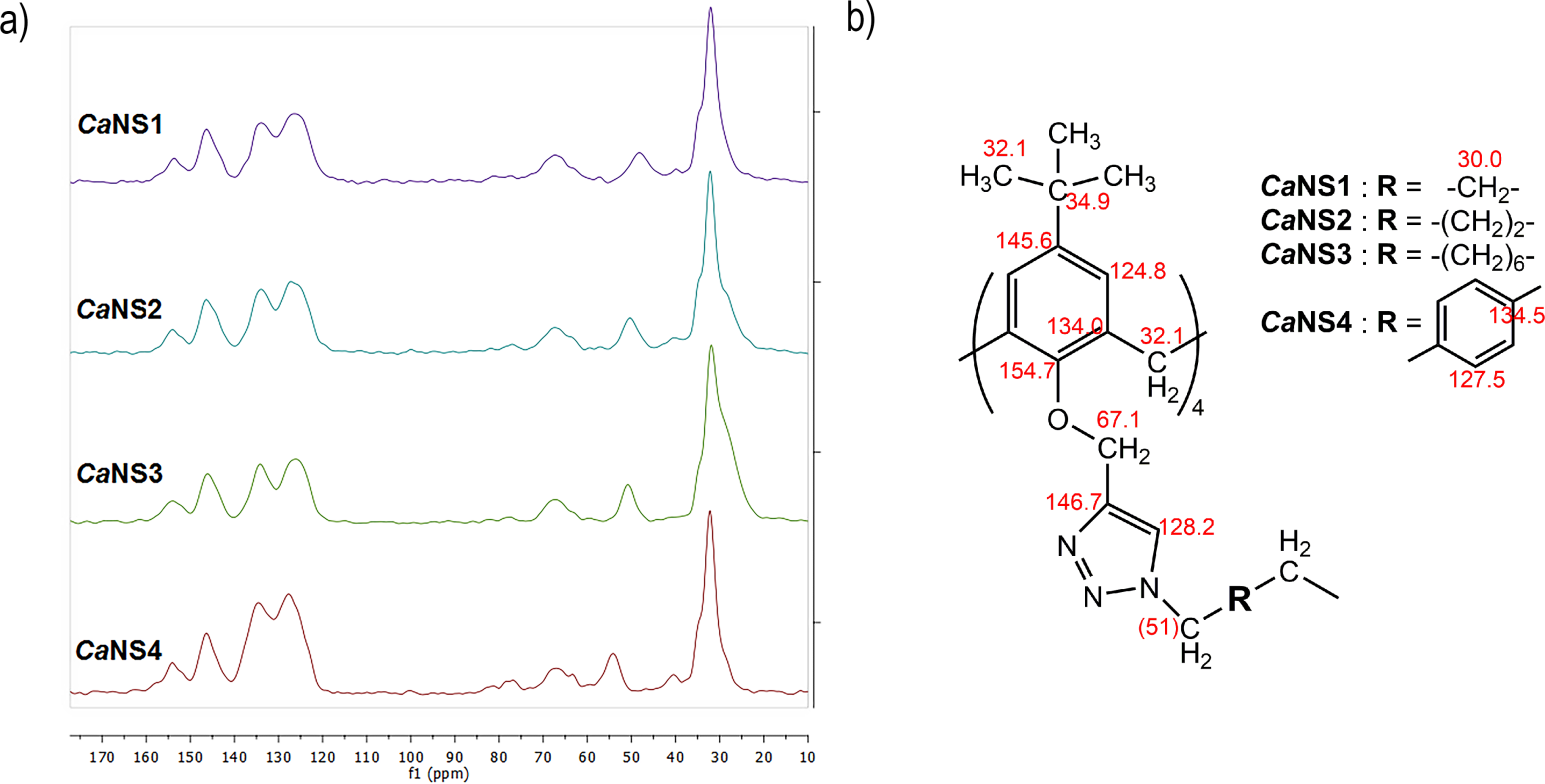
a) ^13^C{^1^H} CP-MAS NMR spectra of *Ca*NSs; b) signal attributions.

The CP-MAS solid-state NMR technique has been proven a versatile and powerful tool for the structural characterization of materials and cross-linked polymers [37] in general, and of NSs in particular. Therefore, it is progressively assuming an importance comparable to that of liquid-state NMR for “small” molecules. The ^13^C{^1^H} CP-MAS spectra of materials *Ca*NS1–*Ca*NS4 are shown synoptically in Figure 2a. The spectra clearly present all the signals expected on the grounds of the structure of the virtual monomer unit depicted in Figure 2b. The attribution can be carried out according to literature reports relevant to *CyCa*NSs.

In the aliphatic C region of the spectrum we first notice an intense signal centered at ca. 32 ppm, clearly constituted by the superimposition of different peaks. This can be cumulatively attributed to the C atoms of the *tert*-butyl groups (both primary and quaternary), the methylene linkers of the calixarene scaffold and, for CaNS1–CaNS3, to the secondary C atoms of the -R- moiety of linker (i.e., the methylene groups that are not directly bound to a triazole ring). Deconvolution analysis of the signal as a sum of Gaussian peaks, enables to individuate three distinct resonances centered at 30.0 ± 0.5, 32.1 ± 0.1 and 34.9 ± 0.1 ppm, which can be tentatively attributed to the secondary, the primary and the quaternary C atoms, respectively. All the spectra also show two other main signals at ca. 51 ± 3 ppm and 67.1 ± 0.2 ppm. The first one can be attributed to the methylene C atoms directly bound to the N1 atom of the triazole ring; its actual position varies significantly depending on the structure of the linker, spanning from 48.2 ppm for *Ca*NS1, to ca. 50.5 ppm for *Ca*NS2 and *Ca*NS3, and to 54.2 ppm for *Ca*NS4. The second main signal, in turn, can be attributed to the methylene C atoms linked to the O atoms of the calixarene scaffold. Further tiny signals can be envisaged at ca. 40, 77 and 81 ppm, which can be tentatively attributed to methylene C atoms linked to unreacted azide groups and to the C_sp_ atoms of unreacted alkyne groups. The latter finding provides further confirmation that the CuAAC reaction cannot run to completion under the conditions for the formation of the polymer network. In the aromatic C region, a cluster of four signals is always present, centered at ca. 127, 134, 146 and 154 ppm, respectively. Even in this case, close inspection of these signals reveals that they are actually given by the superimposition of various Gaussian peaks. The former signal can be cumulatively attributed to the C4 and symmetric atoms of the calixarene scaffold (124.8 ± 1.0 ppm), together with the C5 atom of the triazole ring (128.2 ± 0.7 ppm); the second one to the C1 and symmetric calixarene atoms (134.0 ± 0.5 ppm); the third one cumulatively to the C5 and symmetric calixarene atoms (145.7 ± 0.3 ppm) together with the C4 of the triazole ring (146.3 ± 0.6 ppm); the last one to the C25 and symmetric calixarene atoms (154.0 ± 0.4 ppm). In the case of *Ca*NS4, the resonances of the aromatic C atoms for the aromatic -R- moiety of linker can be envisaged at ca. 127.5 and 134.5 ppm.

As a further remark, it is well known that the CP-MAS technique allows also a semi-quantitative analysis of the signals in the spectrum [11]. It is interesting to notice that along the series *Ca*NS1–*Ca*NS3 the area of the aliphatic signal at ca. 32 ppm increases as compared to cumulative area of the aromatic signals, consistently with the relevant increase of C_sp3_ atoms due to the presence of a different linker. On the other hand, the spectrum of *Ca*NS4 shows an enhanced intensity of the aromatic signals, due to the dibenzyl linker. In the latter case it is also possible to notice that the secondary signals at 40, 77 and 81 ppm are significantly more intense as compared to the other materials. The latter finding appears perfectly consistent with the observation of the fairly intense residual -N_3_ signal in the FTIR spectrum discussed previously, and provides a confirmation of the hypothesis that the rigid linker **A4** causes the CuAAC coupling reaction to proceed up to a lesser extent during the polymer network formation process.

### SEM and DSC characterization

**Figure 3:**
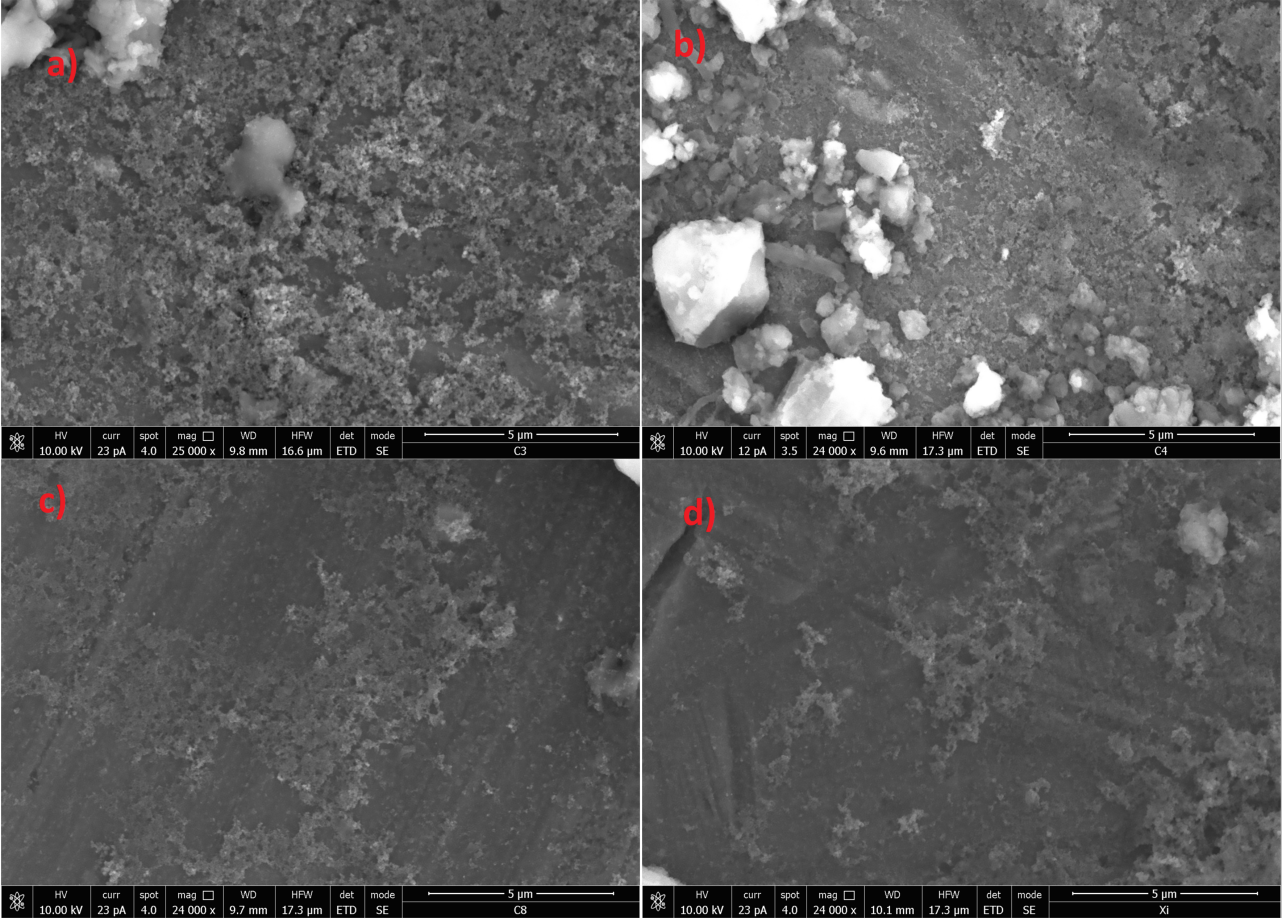
Selection of SEM micrographs for materials for *Ca*NS1 (a), *Ca*NS2 (b), *Ca*NS3 (c) and *Ca*NS4 (d).

Morphological characterization of the materials was accomplished by means of SEM techniques. A selection of micrographs is reported in Figure 3. The materials show very similar apparent features, irrespective of particular linker used. Considering that all products are preliminarily subjected to mechanical grounding and then are passed through a 150 μm sieve, powders show averagely a much finer granulometry. Grains appear quite compact, with a fairly rough surface.

Finally, DSC experiments were performed in order to evaluate the behavior on heating and the thermal stability of the materials. We found out that both the parent *Ca*-OP monomer and the *Ca*NSs materials decompose at ca. 230 °C.

### pH Dependent absorption abilities

The possible pH dependent abilities of *Ca*NSs in sequestering organic species from buffered aqueous solutions were verified, using model *p*-nitroaniline derivatives **1**–**5** and dyes **6**–**10** as the guests. The sequestration abilities of *Ca*NSs were estimated (see Experimental) by equilibrating a given amount of material with a fixed volume of a solution of the guest, and then determining the residual guest concentration by UV–vis spectrophotometry. These experiments were carried out in buffered solutions at different pH values (namely 4.4, 6.7 and 10.7), in order to verify the occurrence of pH-tunable absorption abilities. In analogy with *CyCa*NSs, indeed, possible dependence on pH might arise from the presence of the weakly basic 1,2,3-triazole rings of the linker units, which can supply an overall positive charge density to the polymer lattice upon protonation. A first set of experiments was devoted to *p*-nitroaniline derivatives **1**–**5**. These substrates, indeed, have been considered a class of probe guests of choice for investigating the formation of host–guest inclusion complexes with cyclodextrins [38–41] and calixresorcinarenes [42], because of both their easy accessibility and the fact that their molecular properties can be largely varied though maintaining a fixed chromophore moiety. The results obtained, expressed in terms of percent of guest absorbed, are summarized in Table 1.

**Table 1:** Absorption tests for *Ca*NSs towards *p*-nitroaniline derivatives **1**–**5**.^a^

guest	pH	*Ca*NS1	*Ca*NS2	*Ca*NS3	*Ca*NS4

**1**	4.4	52	38	33	43
	6.7	54	53	38	53
	10.7	51	45	32	60
**2**	4.4	28	67	9	50
	6.7	5	12	7	7
**3**	4.4	50	59	12	71
	6.7	52	72	15	75
**4**	4.4	94	87	86	93
	6.7	96	96	89	98
**5**	6.7	45	50	18	56
	10.7	79	78	28	87

^a^Data are given within a ±3% indetermination.

As we can notice, the parent *p*-nitroaniline (**1**) is absorbed fairly well by *Ca*NSs under neutral conditions, up to an extent comparable with the one reported in literature for the *CyCa*NSs prepared with the largest amount of the calixarene component [11]. Variations in the sequestration abilities towards this guest appear relatively modest on a large pH range (4.4–10.7) for the materials bearing the aliphatic linkers *Ca*NS1–*Ca*NS3, whereas a larger effect is apparent for the material *Ca*NS4 prepared with the rigid aromatic linker. Similarly, only fair variations in the sequestration percentage can be observed on passing from nearly neutral (6.7) to fairly acidic (4.4) pH for hydrophobic guests **3** and **4**. By contrast, sequestration abilities towards the anionic derivative **2** increase significantly on decreasing the pH. This behavior accounts for the occurrence of favorable Coulomb interactions between the guest and the polymer lattice, due to the protonation of the triazole linker units. It is worth noting here that, on the grounds of the p*K*_HA_ value reported in literature (3.18 ± 0.01), **2** is still in its anionic dissociated form at pH 4.4. Similarly, a neat increase of the sequestration percentage occurs, on increasing the pH from 6.7 to 10.7, for the basic diamine derivative **5** (p*K*_BH+_ = 10.19 ± 0.01), which passes from its cationic to its neutral form. A comparison between the substrates shows that an increase in the hydrophobic character of the guest largely favors its sequestration by the materials. This is particularly apparent, for instance, on comparing guests **4** and **5**, both bearing a butyl chain, but differing for the presence in the second one of an additional hydrophilic primary amine group.

In a second series of tests we considered a set of dyes. In particular, we selected probe molecules having diverse structural frameworks, namely an acridinium derivative (toluidine blue, **6**), a diazoic (methyl orange, **7**), a triarylmethane (bromocresol green, **8**), a bis-indole (indigo carmine, **9**) and a naphthalene-bis-diazoic (naphthol blue-black, **10**), in order to evaluate the outcome of molecular size, shape and electric charge. Dyes can be considered suitable and easily detectable models of pollutants [43–47], for the removal of which several techniques have been exploited, spanning from oxidation to photodegradation. Tests were performed only with materials *Ca*NS2 and *Ca*NS4, which appeared on average the best absorbents towards *p*-nitroanilines. The results of the sequestration tests on dyes **6**–**10** are summarized in Table 2.

**Table 2:** Absorption tests for *Ca*NSs towards dyes **6**–**10**.^a^

guest	pH	*Ca*NS2	*Ca*NS4

**6 BT**	4.4	0	16
	6.7	23	56
	10.7	61	93
**7 MA**	4.4	71	41
	6.7	28	18
	10.7	0	3
**8 VBC**	4.4	0	26
	6.7	2	15
	10.7	0	8
**9 CI**	4.4	36	38
	6.7	14	33
	10.7	96	88
**10 NBB**	4.4	51	28
	6.7	41	23

^a^All data are given within a ±3% indetermination.

Consistently with the behavior of ionisable guests **2** and **5**, data indicate a significant dependence of the absorption on pH, which can be partly rationalized in terms of charge interactions. Indeed, absorption of cationic toluidine blue **6** regularly increases on increasing the pH, i.e., on decreasing the possible positive charge on the polymeric lattice, whereas the opposite is found with anionic methyl orange (**7**) and bromocresol green (**8**). However, the anomalous datum for the large indigo carmine dianion **9** at pH 10.7, suggests that other factors may come into play, probably depending on the peculiar size and shape of the guest. It is worth noting that *Ca*NS4 shows better sequestration abilities than *Ca*NS2 towards **6** and **8**. This may be justified admitting that the presence of the aromatic linker unit favors the interaction with the aromatic dye, due to the occurrence of effective π···π interactions with the relatively compact structure of these dyes. On the other hand, the order is reversed for the largest guest **10**, probably because its bulky structure cannot be comfortably accommodated into the relatively stiff pores of the material. Thus, everything considered, the affinity of the guest for *Ca*NSs is confirmed to depend on a fine balance between different factors, namely Coulomb and π···π interactions, and steric effects. The importance of a compromise between these factors for the complexation into calixresorcinarenes has been recently assessed [42]. Moreover, in analogy with the bahavior observed for *Cy*NSs and *CyCa*NSs, such a balance seems significantly affected by the reduced dynamic flexibility of the host monomer unit, which is less prone to optimize its conformation upon the structure of the guest, owing to the hyper-reticulated nature of the material.

## Conclusion

In the present work we report on the synthesis and characterization (FTIR, solid-state NMR, SEM) of a new class of entirely synthetic nanosponge materials based on calixarenes (*Ca*NSs), by reacting a tetrakis(propargyloxy)calix[4]arene with alkyl diazides. The synthesis was accomplished by means of a classical “click” approach, similar to the one used for the synthesis of *CyCa*NSs. However, severer reaction conditions are needed, due to a different number of reactive azide groups as compared, for instance, with the much more effective heptakis(6-azido-6-deoxy)cyclodextrins. The materials obtained have been successfully tested as sequestering agents towards model pollutants such as *p*-nitroaniline derivatives and dyes. Our results indicate that the absorption equilibrium is affected by a combination of hydrophobic effects, Coulomb and π^…^π interactions, as a function of the molecular size and shape. Moreover, significant pH-dependent absorption abilities towards charged guests can be evidenced, due to the possible protonation of the triazole linker subunits.

As a final remark, it is worth noting here that, although the well-known hyper-reticulated polystyrene polymers [48–49] have been occasionally addressed to as “nanosponges” [50], our work reports the first example of entirely synthetic materials based on preorganized and structurally well-defined synthons that are able to function as supramolecular hosts per se. This leads to enhanced absorption performances for our materials in comparison with literature reports on polystyrenes [51–53]. Moreover, the preparation of our materials, unlike hyper-crosslinked polystyrenes [48–49], requires the use of no porogen additive. Hence, our new materials benefit from significant advantages. Considering that polystyrenes have been used also as supporting materials for nanosized inorganic species such as copper oxide [54] or noble metals (Pt [55] and Pd [56]) able to catalyze organic reactions, our nanosponges undoubtedly appear as promising new platforms for the preparation of composite nanocatalysts or functional materials with improved performances.

## Experimental

All the reagents and materials needed were used as purchased (Sigma-Aldrich-Fluka) with no further purification. The tetrakis(propargyloxy)calix[4]arene *Ca*-OP was synthesized according to literature [57]. Similarly, non-commercial *p*-nitroniline derivatives **2**–**5** were prepared according to well assessed procedures [38], by a nucleophilic aromatic displacement reaction between p-fluoronitrobenzene and the proper amine. Alkyl diazides **A1**–**A4** were obtained by a trivial nucleophilic displacement reaction between the proper alkyl dihalide and sozium azide, according to the following procedure. The proper alkyl dihalide (10.0 mmol, namely 2.02 g of 1,3-dibromopropane, or 2.18 g of 1,4-dibromobutane, or 2.72 g of 1,8-dibromooctane, or 2.64 g of 1,4-bis(bromomethyl)benzene) was dissolved in DMF (10 mL) and NaN_3_ (1.95 g, 10.0 mmol) was added. The mixture was kept under magnetic stirring at 55 °C for 48 h. The reaction mixture was then poured into water (50 mL) and extracted thrice with diethyl ether (50 mL each). The organic extracts were dried (brine, then Na_2_SO_4_) and distilled in vacuo (Rotavapor) to afford a crude, which was finally purified by column chromatography (silica, diethyl ether as eluent).

**1,3-Diazidopropane (A1)**. Yield 1.01 g (8.0 mmol, 80%). Yellow oil. IR (liquid): 2095 cm^−1^ (-N_3_); ^1^H NMR (CDCl_3_) δ (ppm): 1.83 (quint, *J* = 6.5 Hz, 2H, -CH_2_-C*H*_2_-CH_2_-), 3.41 (t, *J* = 6.5 Hz, 4H, -CH_2_-N_3_); ESIMS 127.0279 (calcd for C_3_H_6_N_6_ + H^+^, 127.0727).

**1,4-Diazidobutane (A2)**. Yield 1.29 g (9.2 mmol, 92%). Yellow oil. IR (liquid): 2098 cm^−1^ (-N_3_); ^1^H NMR (CDCl_3_) δ (ppm): 1.68 (m, 4H, -CH_2_-C*H*_2_-), 3.33 (m, 4H, -CH_2_-N_3_); ESIMS 141.0891 (calcd for C_4_H_8_N_6_ + H^+^, 141.0883).

**1,8-Diazidooctane (A3)**. Yield 1.67 g (8.5 mmol, 85%). Pale yellow oil. IR (liquid): 2096 cm^−1^ (-N_3_); ^1^H NMR (CDCl_3_) δ (ppm): 1.34, 1.38 (2m, 8H, -CH_2_-), 1.60 (quint, *J* = 6.5 Hz, 4H, -C*H*_2_-CH_2_-N_3_), 3.26 (t, *J* = 6.5 Hz, 4H, -CH_2_-N_3_); ESIMS 197.1521 (calcd for C_8_H_16_N_6_ + H^+^, 197.1509).

**1,4-Bis(azidomethyl)benzene (A4)**. Yield 1.77 g (9.4 mmol, 94%). Yellow oil. IR (liquid): 2099 cm^−1^ (-N_3_); ^1^H NMR (CDCl_3_) δ (ppm): 4.36 (s, 4H, -CH_2_-*N*_3_-), 7.35 (s, 4H, -C_6_H_4_-); ESIMS 189.0878 (calcd for C_8_H_8_N_6_ + H^+^, 189.0883).

UV–vis spectra were recorded on a Bruker DU 800 spectrophotometer, equipped with a Peltier thermostatic apparatus. FTIR spectra (nujol) were recorded on an Agilent Technologies Cary 630 FTIR spectrometer. NMR spectra were acquired on a Bruker Avance II 400 MHz instrument, equipped (for solid-state experiments) with a 15 kHz rotating MAS probe. High-resolution ESIMS mass spectra were acquired in positive mode on an AGILENT Technologies 6540 UHD Accurate Mass Q-TOF LC–MS apparatus (1 kV nozzle voltage, 175 V fragmentor voltage). SEM micrographs were acquired on a FEG-SEM FEI versa 3D microscope. The acceleration voltage was 10 kV. Tiny amounts of powdered samples were dispersed in ethanol. Two drops were deposited on the stub and let evaporating. Micrographs were directly acquired without metalation. DSC determinations were performed on a DSC Q200 calorimeter interfaced to a TA Thermal Analyst 3100 controller connected to an RCS90 cooling system. Heating/cooling cycles in the range 25–300 °C were accomplished under N_2_ stream, at a rate of 20 °C/min. Samples of the materials (weighed in T-zero aluminum pans) were tested to verify their decomposition after DSC experiment.

### Synthesis of *Ca*NSs

In a dark screw-cap glass vial the starting calixarene Ca-OP (200.0 mg, 0.25 mmol) was placed, with CuSO_4_·5H_2_O (40.0 mg, 0.16 mmol) and sodium ascorbate (62.0 mg, 0.31 mmol) were added; then the alkyl diazide was added (0.5 mmol, namely 63.0 mg of **A1**, or 70.0 mg of **A2**, or 98.0 mg of **A3**, or 94.0 mg of **A4**), and the mixture was dissolved with DMSO (2 mL). The system was kept under magnetic stirring at 65 °C for 90 h). The reaction crude was then poured into water (60 mL), sonicated for a few minutes and centrifuged at 5500 rpm for 15 min. The solid was decanted, suspended in water (50 mL) sonicated for 10 min and centrifuged again. The solid was then subjected to two further suspension–sonication–centrifugation washing cycles using methanol (50 mL) and diethyl ether (50 mL), respectively; in which the product materials are insoluble. The product was finally recovered by filtration in vacuo, grinded, passed through a 150 μm sieve and desiccated in vacuo over P_2_O_5_ at 50 °C. Yields 250 mg for *Ca*NS1, 234 mg for *Ca*NS2, 263 mg for *Ca*NS3, 278 mg for *Ca*NS4. It is worth noting here that, after drying and sieving, the solid products showed no appreciable swelling in aqueous solution.

### Absorption tests

Stock solutions 50 μM of the different guests were prepared in aqueous buffers at the desired pH values. Samples were prepared by mixing 2 mL of guest solution with a carefully weighed amount (4.00 ± 0.05 mg) of material. The samples were shaken at room temperature for 90 min, and then centrifuged for 15 min at 5500 rpm. The supernatant liquor was carefully pipetted after centrifugation, and then the percent amount of guest left in solution at equilibrium was simply estimated by means of UV–vis spectrophotometry, comparing the absorbances of the starting and final solutions.
